# Surgery and anaesthesia during the heroic age of Antarctic exploration (1895-1922)

**DOI:** 10.1136/bmj.f7242

**Published:** 2013-12-17

**Authors:** H R Guly

**Affiliations:** 1 Emergency Medicine and British Antarctic Survey Medical Unit, Derriford Hospital, Plymouth PL6 8DH, UK

## Abstract

During the heroic age of Antarctic exploration, general anaesthesia was given on at least 11 occasions. This paper describes some of the surgery procedures performed, including on the doctors themselves

**Figure 2:**
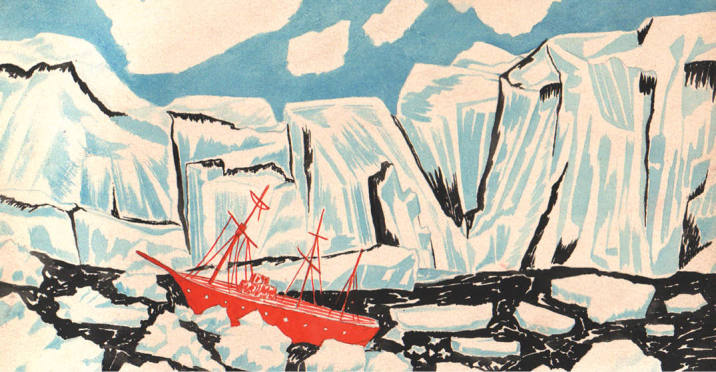
TATIANA GLEBOVA, FROM INSIDE THE RAINBOW, REDSTONE PRESS

During the heroic age of Antarctic exploration, there were 18 exploring and scientific expeditions to the Antarctic: 14 of these took doctors who performed at least 11 surgical procedures under general anaesthesia (table[Table tbl1]).^1^ ^2^ ^3^ ^4^ ^5^ ^6^ ^7^ ^8^ ^9^ ^10^ ^11^ ^12^ ^13^ ^14^ Other surgery is described with no mention of the type of anaesthesia. This paper describes, for historical interest, some of the operations performed.

**Table tbl1:** Surgery performed under general anaesthesia

Expedition	Operation	Surgeon	Anaesthetist	Anaesthetic	Notes	Reference
**Surgery to people**						
Discovery expedition (1901-04)	Dental extraction for abscess	Reginald Koettlitz	Edward Wilson	Ether	—	1
First German Antarctic expedition (1901-03)	Drainage of prostatic abscess	Hans Gazert	Non-medical person	Chloroform	—	2
Nimrod expedition (1907-09)	Enucleation of eye for trauma	Eric Marshall	Alister Forbes Mackay	Chloroform	—	3,4
Nimrod expedition	Amputation of toe for frostbite	Eric Marshall	Alister Forbes Mackay	Not stated	—	5
*Endurance* expedition (1914-17)	Amputation of toes for frostbite	James McIlroy	Alexander Macklin	Chloroform	—	6-8
*Pourquoi Pas?* expedition (1908-10)	Amputation of fingers following trauma	Jacques Liouville	Ernest Gourdon (a medical student)	Chloroform	Patient was a whaler, not an expedition member	9
*Pourquoi Pas?* expedition	Repair of foot wound involving joint	Probably Jacques Liouville	Probably Ernest Gourdon	Chloroform	—	10
Second German Antarctic expedition	Appendicectomy	Wilhelm Goeldel	Non-medical person	Ether	Patient was Dr Ludwig Kohl	11
Australasian Antarctic expedition (1911-14)	Dental extraction	Leslie Whetter	Leslie Whetter	Chloroform	Patient was Dr Archibald McLean	12
**Surgery to dogs**						
Australasian Antarctic expedition	Repair of neck wound	Archibald McLean	Leslie Whetter	Chloroform	—	13
*Endurance* expedition	Repair of laceration around eye	Probably James McIlroy	Probably Alexander Macklin	Chloroform	—	14

## Surgery under general anaesthesia

The first general anaesthetic recorded as having been given on an Antarctic expedition was during Robert Scott’s Discovery expedition (1901-04). The chief engineer required a dental extraction for an abscess and anaesthetist Edward Wilson described:

“He will not undergo any treatment at all—simply will not stand the pain of having his gum lanced or a stump drawn. So . . . I gave him ether, and [surgeon Reginald] Koettlitz drew a tooth and we made a job of it. He was under nearly 25 minutes and the whole thing was very successful. He knew absolutely nothing of what had been done, went off almost immediately. There was much amusement on deck over his loud and amusing songs and unparliamentary remarks as he was recovering from the effects of the ether. He was only sick once, and had practically no after effects.”^1^

One wonders whether the patient would have been reassured by Wilson’s further note: “I gave it him on the practical experience I had gained by receiving it myself last year” (when having an axillary abscess incised).^1^

The worst conditions for surgery undoubtedly occurred during Ernest Shackleton’s second expedition (on the *Endurance* in 1914-17). After the ship had been trapped in the pack ice and eventually crushed, the men were forced to camp on the ice for six months. When this ice broke up, they sailed and rowed for a week in small boats to Elephant Island where two upturned boats were converted into huts in which the 22 men lived in squalor (fig[Fig fig1]). Percy Blackborrow, who had been a stowaway but after being discovered was incorporated into the ship’s crew, developed frostbite on the boat journey and his toes became gangrenous. A month later, it was decided that he needed surgery, which anaesthetist Alexander Macklin described in his diary:

“Today [surgeon James] McIlroy operated on Blackboro’, amputating all the toes of the left foot. I gave CHCl_3_: he took his anaesthetic very well and was not at all sick afterwards. We managed to sterilise instruments pretty well by using a primus and hoosh-pot [cooking pot]. We heated up water over the stove and [photographer Frank] Hurley took charge of the fire and succeeded in keeping it going without making any smoke in the hut. We managed to get the temperature up to 80° [Fahrenheit; 27°C] and the CHCl_3_ vaporised splendidly. We had only 8 oz [227 g] of chloroform, but although the operation lasted 55 minutes, I only used an ounce . . . The operating table consisted of packing cases, and I had another one for a stool. We had no sterilised overalls to get into: we merely stripped to our vests . . . Blackboro’ was soon round from the anaesthetic and asked for that now rare luxury—a cigarette.”^6^

**Figure fig1:**
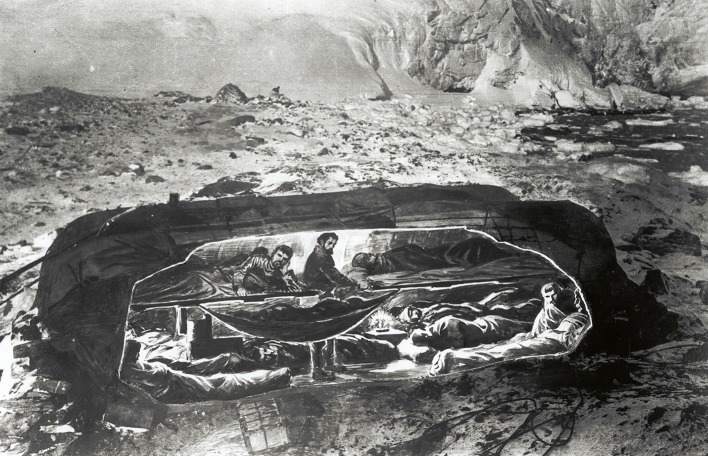
The upturned boat on Elephant Island that served as the operating theatre for the amputation of Blackborrow’s toes

Leonard Hussey, a meteorologist but who qualified late as a doctor, gave more information: “The patient’s head was placed as near to our little oil-drum stove as was possible and the stove was then stoked up with lavish supplies of seal-blubber. This helped the chloroform to volatilise, which otherwise would have been difficult owing to the cold.”^7^ Hurley added a bit more colour, describing “the feeble glimmer of blubber lamps” and “[maintaining] the temperature of the ‘theatre’ at 50° by stoking up the bogie-fire with penguin skins.”^8^ The unsanitary conditions were added to by the presence of a patient with a discharging buttock abscess who was too sick to be moved.

Modern advice would be to wait much longer before amputating in frostbite, but amputation (for open fractures as well as for frostbite) was resorted to at a much earlier stage in the pre-antibiotic era, because of the life threatening consequences of infection.

Only one abdominal operation seems to have been performed. The second German Antarctic expedition (1911-13) sailed with two doctors and one of them (Ludwig Kohl) developed appendicitis. Expedition leader Wilhelm Filchner reports: “Assisted by the captain, the first officer . . . and the steward, his colleague [Wilhelm Goeldel] immediately started the operation; it took 90 minutes . . . Occasionally one of the . . . [members of the mess] would peep down through the skylight into the operating room, in order to report on the progress of the work which was being carried out on the dining table . . . Fortunately the sea was mirror-smooth and calm throughout the operation. The engine was stopped during this period.”^2^ Kohl got up two days after his operation, but when the ship reached South Georgia he left the expedition to convalesce.

On Shackleton’s Nimrod expedition (1907-09), second mate Æneas Mackintosh was struck in the eye by a hook while the ship was unloading stores. Surgeon Eric Marshall’s diary says that he “examined him and found what appeared to be a portion of retina protruding through eye. [Ernest] Joyce tells me that when he fell he saw lens lying on his cheek. Kept him under, 1st atropine and cocaine, until 2.30, when assisted by [Alister Forbes] Mackay and [ship surgeon Rupert] Michell we gave him chloroform, with leave to act as we thought right. Found eye collapsed, cornea torn right across centre, lens absent, much of the vitreous humour had escaped and retina torn. We unanimously decided to excise eye. Operation was successfully performed although circumstances adverse owing to lack of space, appliances etc.”^3^ A typed transcript of the diary (presumably by Marshall himself) gives additional details that are not in his original: “one pair of curved scissors only were available. I made . . . hooks and retractor from rigging wire. Mackay’s Edinburgh method of giving anaesthetics with a towel added to the difficulties. Mackintosh lay on the cabin floor, on which we knelt, and the only light was an oil lamp.”^4^ Interestingly, Marshall had been qualified about 18 months, during which six months had been spent travelling to the Antarctic: how many modern doctors, much longer qualified than Marshall, would contemplate doing such surgery?

## Local anaesthesia

Much local anaesthetic was obviously used—Edward Atkinson of Scott’s Terra Nova expedition (1910-13) said that more would have been useful, but I have found no mention of its use other than for one dental procedure^12^ (and the topical use of cocaine in the eye).

One operation, on the Discovery expedition, was performed under what was, presumably, ethyl chloride spray, although this was not on the list of medical equipment supplied to that expedition.^15^ Physicist Louis Bernacchi described:

“When [first lieutenant Charles] Royds was operated upon for a cyst on his cheek, the general reaction was one of pleasurable interest rather than sympathy for the unfortunate victim. Dr [Reginald] Koettlitz, nothing loath to perform the first operation in Antarctica, gladly prepared for the event. The wardroom table became the operating table. I volunteered as nurse, and rolled up my sleeves to play the part convincingly, while Koettlitz brought from their hiding-places a formidable array of knives, pincers, scissors, lint, gauze and bandages, explaining ghoulishly the exact function of each. Armitage took charge of the phial of patent freezing mixture, and the rest of the wardroom gathered round. The effort at first was not a success, for the freezing mixture functioned so thoroughly that the knife would not penetrate the skin, and while we waited for it to thaw a little, all joined in terrifyingly reassuring remarks to the patient. Again the knife was applied, and this time, to our intense satisfaction, blood flowed. Our questions as to whether it hurt or not brought a most emphatic ‘Yes’. But the cyst was removed and the cheek stitched up, and Royds was distinguished for the rest of his life by a diminutive scar, a record of the first surgical operation performed in Antarctica.”^16^

In summary, conditions for surgery in the Antarctic during this era were makeshift and far from ideal. Much local anaesthetic was used but it is frustrating that we do not have more details of these cases. Two general anaesthetics were given to expedition doctors. Therefore, when planning medical care for an expedition—even now—consideration needs to be given to the fact that a doctor is as likely to become ill or injured as any other expedition member.

## Notes

This paper is part of a bigger project on medicine during the heroic age of Antarctic exploration, based on published and unpublished expedition reports, diaries, equipment lists, and medical reports. This was made possible by a research grant from the Wellcome Trust.

Provenance and peer review: Not commissioned; externally peer reviewed.

## Notes

Cite this as: *BMJ* 2013;347:f7242
